# Mondo: integrating disease terminology across communities

**DOI:** 10.1093/genetics/iyaf215

**Published:** 2025-10-06

**Authors:** Nicole A Vasilevsky, Sabrina Toro, Nicolas Matentzoglu, Joseph E Flack, Kathleen R Mullen, Harshad Hegde, Sarah Gehrke, Patricia L Whetzel, Yousif Shwetar, Nomi L Harris, Mee S Ngu, Gioconda L Alyea, Megan S Kane, Paola Roncaglia, Eric Sid, Courtney L Thaxton, Valerie Wood, Roshini S Abraham, Maria Isabel Achatz, Pamela Ajuyah, Joanna S Amberger, Lawrence Babb, Jasmine Baker, James P Balhoff, Jonathan S Berg, Amol Bhalla, Xavier Bofill-De Ros, Ian R Braun, Eleanor C Broeren, Blake K Byer, Alicia B Byrne, Tiffany J Callahan, Leigh C Carmody, Lauren E Chan, Amanda R Clause, Julie S Cohen, Marcello DeLuca, Natalie T Deuitch, May Flowers, Jamie Fraser, Toyofumi Fujiwara, Vanessa Gitau, Jennifer L Goldstein, Dylan Gration, Tudor Groza, Benjamin M Gyori, William Hankey, Jason A Hilton, Daniel S Himmelstein, Stephanie S Hong, Charles T Hoyt, Robert Huether, Eric Hurwitz, Julius O B Jacobsen, Atsuo Kikuchi, Sebastian Köhler, Daniel R Korn, David Lagorce, Bryan J Laraway, Jane Y Li, Adriana J Malheiro, James McLaughlin, Birgit H M Meldal, Shruthi Mohan, Sierra A T Moxon, Monica C Munoz-Torres, Tristan H Nelson, Frank W Nicholas, David Ochoa, Daniel Olson, Tudor I Oprea, Tomiko T Oskotsky, David Osumi-Sutherland, Kelley Paris, Helen E Parkinson, Zoë M Pendlington, Xiao P Peng, Amy Pizzino, Sharon E Plon, Bradford C Powell, Julie C Ratliff, Heidi L Rehm, Lyubov Remennik, Erin R Riggs, Sean Roberts, Peter N Robinson, Justyne E Ross, Kevin Schaper, Brian M Schilder, Johanna L Schmidt, Elliott W Sharp, Morgan N Similuk, Damian Smedley, Tam P Sneddon, Rachel Sparks, Ray Stefancsik, Gregory S Stupp, Shilpa Sundar, Terue Takatsuki, Imke Tammen, Kezang C Tshering, Deepak R Unni, Eloise Valasek, Adeline Vanderver, Alex H Wagner, Ryan F Webb, Danielle Welter, Doron Yaya-Stupp, Andreas Zankl, Xingmin Aaron Zhang, Julie A McMurry, Christopher G Chute, Ada Hamosh, Christopher J Mungall, Melissa A Haendel

**Affiliations:** Data Collaboration Center, Critical Path Institute, Tucson, AZ 85718, United States; Department of Genetics, University of North Carolina at Chapel Hill, Chapel Hill, NC 27514, United States; Semanticly, Athens 10563, Greece; School of Medicine, Johns Hopkins University, Raleigh, NC 27617, United States; Department of Genetics, University of North Carolina at Chapel Hill, Chapel Hill, NC 27514, United States; Lawrence Berkeley National Laboratory, EGSB, Berkeley, CA 94720, United States; Department of Genetics, University of North Carolina at Chapel Hill, Chapel Hill, NC 27599, United States; Department of Genetics, University of North Carolina at Chapel Hill, Chapel Hill, NC 27599, United States; Joint Department of Biomedical Engineering, University of North Carolina at Chapel Hill, Chapel Hill, NC 27519, United States; Lawrence Berkeley National Laboratory, EGSB, Berkeley, CA 94720, United States; Department of Genetics, University of North Carolina at Chapel Hill, Chapel Hill, NC 27599, United States; National Organization for Rare Disorders, Medical Affairs, Quincy, MA 02169, United States; National Library of Medicine, National Institutes of Health, National Center for Biotechnology Information, Bethesda, MD 20894, United States; Elsevier SciBite, Curation Team, Hinxton, Cambridge CB10 1DR, United Kingdom; NCATS Division of Rare Diseases Research Innovation, National Institutes of Health, Bethesda, WA 98332, United States; Department of Genetics, University of North Carolina at Chapel Hill, Chapel Hill, NC 27599, United States; Department of Biochemistry, University of Cambridge, Cambridge CB2 1QW, United Kingdom; Pathology and Laboratory Medicine, Nationwide Children's Hospital, Columbus, OH 43205, United States; Department of Oncologia, Hospital Sirio-Libanes, Sao Paulo, SP 01308-901, Brazil; Program in Medical and Population Genetics, Broad Institute of MIT and Harvard, Cambridge, MA 02142, United States; Department of Medical Genetics, Johns Hopkins University School of Medicine, Baltimore, MD 21287, United States; Medical and Population Genetics, Broad Institute of MIT and Harvard, Cambridge, MA 02142, United States; Department of Pediatrics-Oncology, Baylor College of Medicine, Houston, TX 77030, United States; RENCI, University of North Carolina at Chapel Hill, Chapel Hill, NC 27517, United States; Department of Genetics, University of North Carolina at Chapel Hill, Chapel Hill, NC 27599, United States; Global Clinical Services/Clinical Informatics, Intelligent Medical Objects, Rosemont, IL 60018, United States; Department of Molecular Biology and Genetics, Aarhus University, Aarhus 8000, Denmark; Data Collaboration Center, Critical Path Institute, Tucson, AZ 85718, United States; Medical and Population Genetics, Broad Institute of MIT and Harvard, Cambridge, MA 02142, United States; Department of Genetics, University of North Carolina at Chapel Hill, Chapel Hill, NC 27514, United States; Program in Medical and Population Genetics, Broad Institute of MIT and Harvard, Boston, MA 02140, United States; IBM Research Accelerated Materials Discovery, San Jose, CA 95120, United States; Jackson Laboratory, Farmington, CT 06730, United States; Department of Pediatrics, University of Chicago, Chicago, IL 60637, United States; Department of Neurology, Washington University in St. Louis, St. Louis, MO 63130, United States; Neurology and Developmental Medicine, Kennedy Krieger Institute, Baltimore, MD 21205, United States; Department of Chemical Biology and Medicinal Chemistry, University of North Carolina at Chapel Hill, Chapel Hill, NC 27599, United States; Translational and Functional Genomics Branch, National Human Genome Research Institute, Bethesda, MD 20892, United States; Department of Genetics, University of North Carolina at Chapel Hill, Chapel Hill, NC 27560, United States; Children's National Hospital: Washington, D.C., Rare Disease Institute, Washington, DC 20010, United States; Database Center for Life Science, Research Organization of Information and Systems, Kashiwa, Chiba 277-0871, Japan; Medical and Population Genetics, Broad Institute of MIT and Harvard, Cambridge, MA 02142, United States; Department of Genetics, University of North Carolina at Chapel Hill, Chapel Hill, NC 27510, United States; Western Australian Register of Developmental Anomalies, King Edward Memorial Hospital, Perth, Western Australia 6008, Australia; Agency for Science, Technology and Research (A*STAR), Bioinformatics Institute (BII), Singapore 138671, Singapore; Khoury College of Computer Sciences, Northeastern University, Boston, MA 02115 02445, United States; Department of Genetics, University of North Carolina at Chapel Hill, Chapel Hill, NC 27514, United States; Department of Biomedical Data Science, Stanford University School of Medicine, Stanford, CA 94305, United States; RadOverlay, Lebanon, NH 03766, United States; General Internal Medicine, BIDS Section, John Hopkins University School of Medicine, Baltimore, MD 21205, United States; RWTH Aachen University, Institute of Inorganic Chemistry, Aachen, NRW 52074, Germany; Department of Bioinformatics, Tempus AI, Chicago, IL 60654, United States; Department of Genetics, University of North Carolina at Chapel Hill, Chapel Hill, NC 27514, United States; Queen Mary University of London, William Harvey Research Institute, London EC1M 6BQ, United Kingdom; Department of Pediatrics, Tohoku University Graduate School of Medicine, Sendai, Miyagi 980-8574, Japan; Ada Health GmbH, AI, Berlin 10179, Germany; Department of Genetics, University of North Carolina at Chapel Hill, Chapel Hill, NC 27514, United States; INSERM, US14, PARIS, Paris 75014, France; Department of Genetics, University of North Carolina at Chapel Hill, Chapel Hill, NC 27599, United States; Every Cure, Tech Team, Philadelphia, PA 19104, United States; National Library of Medicine, National Institutes of Health, National Center for Biotechnology Information, Bethesda, MD 20894, United States; EMBL-EBI, Samples, Phenotypes, and Ontologies (SPOT), Hinxton CB10 1SA, United Kingdom; Pfizer UK, Pfizer Digital, Tadworth, Surrey KT20 7NS, United Kingdom; Department of Pathology, Duke University Health System, Durham, NC 27705, United States; Lawrence Berkeley National Laboratory, EGSB, Berkeley, CA 94720, United States; Department of Biomedical Informatics, University of Colorado Anschutz Medical Campus, Aurora, CO 80045, United States; Department of Developmental Medicine, Geisinger, Danville, PA 17822, United States; Sydney School of Veterinary Science, University of Sydney, Sydney, NSW 2006, Australia; EMBL-EBI, Open Targets, Hinxton, Cambridgeshire CB10 1SD, United Kingdom; Ontologies, Standards and Metadata, Critical Path Institute, Tucson, AZ 85715, United States; Expert Systems Inc., San Diego, CA 92130, United States; Division of Clinical Informatics and Digital Transformation, University of California San Francisco, San Francisco, CA 94143, United States; EMBL-EBI, Samples, Phenotypes and Ontologies, Hinxton, Cambridgeshire CB10 1SD, United Kingdom; Medical and Population Genetics, Broad Institute of MIT and Harvard, Cambridge, MA 02142, United States; EMBL-EBI, Knowledge Management Section, European Bioinformatics Institute, Hinxton CB10 1SD, United Kingdom; EMBL-EBI, Knowledge Management Section, European Bioinformatics Institute, Hinxton CB10 1SD, United Kingdom; Department of Pediatrics/Genetics, Montefiore Medical Center/Albert Einstein College of Medicine, Bronx, NY 10467, United States; Department of Neurology, Children's Hospital of Philadelphia, Philadelphia, PA 19104, United States; Department of Pediatrics, Baylor College of Medicine, Houston, TX 77030, United States; Department of Genetics, University of North Carolina at Chapel Hill, Chapel Hill, NC 27599, United States; Medical and Population Genetics/Translational Genomics Group, Broad Institute of MIT and Harvard, Boston, MA 02134, United States; Center for Genomic Medicine; Medical and Population Genetics, Massachusetts General Hospital; Broad Institute or MIT and Harvard, Boston, MA 02114, United States; CBIIT, National Cancer Institute (NCI), Bethesda, MD 20850, United States; Department of Developmental Medicine, Geisinger, Danville, PA 17822, United States; National Organization for Rare Disorders, Information Technology, Woodbury, CT 06798, United States; Charité Universitätsmedizin Berlin, Berlin Institute of Health, Berlin 10117, Germany; Department of Genetics, University of North Carolina at Chapel Hill, Chapel Hill, NC 27707, United States; Department of Genetics, University of North Carolina at Chapel Hill, Chapel Hill, NC 27599, United States; Quantitative Biology, Cold Spring Harbor Laboratory, Cold Spring Harbor, NY 11724, United States; Department of Pediatrics, Baylor College of Medicine, Houston, TX 77030, United States; Every Cure, Medical Team, Philadelphia, PA 19104, United States; Division of Intramural Research, National Institute of Allergy and Infectious Diseases, National Institutes of Health, Bethesda, MD 20892, United States; Queen Mary University of London, William Harvey Research Institute, London EC1M 6BQ, United Kingdom; Department of Pathology and Laboratory Medicine/Department of Genetics, University of North Carolina at Chapel Hill, Chapel Hill, NC 27514, United States; National Institutes of Health, National Institute of Allergy & Infectious Diseases, Bethesda, MD 20892, United States; EMBL-EBI, Knowledge Management Section, European Bioinformatics Institute, Hinxton CB10 1SD, United Kingdom; Department of Integrative Structural and Computational Biology, Scripps Research Institute, La Jolla, CA 92037, United States; Department of Genetics, University of North Carolina at Chapel Hill, Chapel Hill, NC 27514, United States; Database Center for Life Science Joint Support-Center for Data Science Research Research Organization of Information and Systems, Kashiwa, Chiba 277-0871, Japan; Sydney School of Veterinary Science, The University of Sydney, Camden, NSW 2570, Australia; Translational Genomics Group, Broad Institute of MIT and Harvard, Cambridge, MA 02142, United States; Personalized Health Informatics Group, SIB Swiss Institute of Bioinformatics, Basel 4051, Switzerland; Clinical Epidemiology, The Lady Davis Institute for Medical Research, Montreal, Quebec H3T 1E2, Canada; Department of Pediatrics, Baylor College of Medicine, Houston, TX 77030, United States; Institute for Genomic Medicine, Nationwide Children's Hospital, Columbus, OH 43215, United States; Program in Medical and Population Genetics, Broad Institute of MIT and Harvard, Cambridge, MA 02142, United States; Luxembourg National Data Service, LNDS, Esch-sur-Alzette 4362, Luxembourg; Department of Developmental Biology and Cancer Research, The Institute for Medical Research Israel-Canada, The Hebrew University of Jerusalem, Jerusalem 9112001, Israel; Faculty of Medicine and Health, The University of Sydney, Sydney, NSW 2050, Australia; The Jackson Laboratory for Genomic Medicine, Farmington, CT 06032, United States; Department of Genetics, University of North Carolina at Chapel Hill, Chapel Hill, NC 27514, United States; Schools of Medicine, Public Health, and Nursing, Johns Hopkins University, Baltimore, MD 21287, United States; Department of Genetic Medicine, Johns Hopkins University, Baltimore, MD 21287-4922, United States; Lawrence Berkeley National Laboratory, EGSB, Berkeley, CA 94720, United States; Department of Genetics, University of North Carolina at Chapel Hill, Chapel Hill, NC 27514, United States

**Keywords:** disease integration, rare disease, disease ontology, disease classification, biomedical informatics, disease terminology, community-driven ontology

## Abstract

Precision medicine aims to enhance diagnosis, treatment, and prognosis by integrating multimodal data at the point of care. However, challenges arise due to the vast number of diseases, differing methods of classification, and conflicting terminological coding systems and practices used to represent molecular definitions of disease. This lack of interoperability artificially constrains the potential for diagnosis, clinical decision support, care outcome analysis, as well as data linkage across research domains to support the development or repurposing of therapeutics. There is a clear and pressing need for a unified system for managing disease entities⁠—including identifiers, synonyms, and definitions. To address these issues, we created the Mondo disease ontology—a community-driven, open-source, unified disease classification system that harmonizes diverse terminologies into a consistent, computable framework. Mondo integrates key medical and biomedical terminologies, including Online Mendelian Inheritance in Man (OMIM), Orphanet, Medical Subject Headings (MeSH), National Cancer Institute Thesaurus (NCIt), and more, to provide a comprehensive and accurate representation of disease concepts with fully provenanced and attributed links back to the sources. Mondo can be used as the handle for curation of gene–disease associations utilized in diagnostic applications, research applications such as computational phenotyping, and in clinical coding systems in clinical decision support by pointing the clinician to the numerous knowledge resources linked to the Mondo identifier. Mondo's community-centric approach, stewarded by the Monarch Initiative's expertise in ontologies, ensures that the ontology remains adaptable to the evolving needs of biomedical research and clinical communities, as well as the knowledge providers.

## Introduction: the need for a community-driven disease terminology

**Fig. 1.**
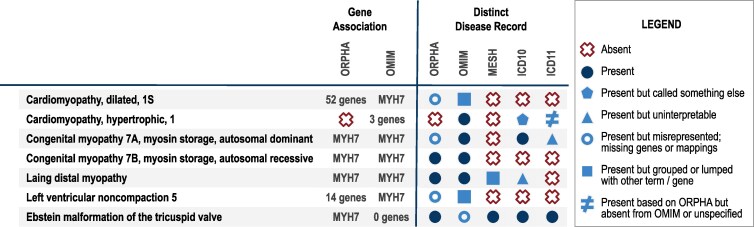
Example of inconsistent disease definitions across terminology resources. MYH7 is a gene associated with multiple diseases and is identified with distinct disease records across various expert community resources. Because the scope and audience of each disease terminology resource vary, the disease records often show conflicting information when they are compared. This lack of harmonization hinders the utility of the resources for use in clinical diagnostics. Mondo addresses this long-standing challenge so expertise and knowledge of all disease resources are easily available to the medical community (status in January 2025).

In the past decade, there have been major advances in computational approaches to disease diagnosis and care management. However, the reference data on which these tools depend are not only heterogeneous and disaggregated, but also growing and ever-changing. While standard terminologies and knowledge sources such as the Online Mendelian Inheritance in Man (OMIM) (Amberger and Hamosh 2017) and Orphanet (Slebodnik 2009; Wakap et al. 2020) have made important strides in standardizing rare disease terminology, differences in scope, structure, and interpretation across these resources persist. Additionally, dozens of terminological disease resources used for research and clinical applications exist (Haendel et al. 2018; Vasilevsky, n.d.), including those for Mendelian diseases, common diseases, rare diseases, cancer, infectious diseases, and others that are more comprehensive and broad (Rogers 1963; Sioutos et al. 2007; Slebodnik 2009; Amberger and Hamosh 2017; Bello et al. 2018). Scope and classification are just two of the ways these resources differ: additional differences include disease naming conventions, synonym encoding, and cross-references. As a result, each terminology has different strengths and weaknesses, and there is often significant overlap between the resources (Richesson and Krischer 2007; Kamdar, Tudorache, and Musen 2017). To mitigate the resulting problem with integrating data across resources, mappings have been developed to connect corresponding disease concepts. However, the correspondence (mapping) among individual concepts is often established through text-matching, but this can be misleading; for example, the terms “Muscular pseudohypertrophy-hypothyroidism syndrome” in Orphanet (Orphanet:2349, see also https://www.orpha.net/en/disease/detail/2349) (Mangaraj and Sethy 2014) and “B-cell immunodeficiency, distal limb anomalies, and urogenital malformations” in OMIM (OMIM:609296) (Hügle et al. 2011) both have the exact synonym “Hoffman syndrome”, but they are entirely different diseases. Mappings are often represented as “cross-references” in biomedical ontologies, but the relationship between the two terms can be nonexact, incorrect, out-of-date, obsolete, or otherwise not clearly defined. For example, the Disease Ontology (DO) concept DOID:8923 “skin melanoma” cross-references both OMIM:608035 “melanoma, cutaneous malignant, susceptibility to, 4” and OMIM:612263 “melanoma, cutaneous malignant, susceptibility to, 7” (Human Disease Ontology Release v2022-03-02 n.d.), which are two different types of susceptibility rather than types of melanoma. Therefore, the resulting integration across disease resources is often incomplete, inconsistent, and too ambiguous for the precision needs of diagnostics or research (Fig. 1). A closely related problem to data integration is the consistent and granular coding of rare diseases in electronic health records (EHRs). Via partnership with IMO Health (imohealth.com) and Epic Systems (epic.com), for example, a subset of Mondo rare disease codes, which integrate rare disease concepts from across different terminologies, are being deployed into healthcare systems and will allow clinicians to easily navigate to the correct content in expert knowledge sources such as OMIM and Orphanet.

A community-driven approach to developing a disease ontology is essential because it brings together broad expertise across multiple disciplines, ensuring the ontology is comprehensive, accurate, and reflective of different clinical, technical, and biological perspectives. This collaborative model allows for rapid updates, incorporating new discoveries and clinical guidelines in real-time, while also promoting global standardization of disease terms to facilitate data integration across research initiatives. By involving global stakeholders, a community-driven ontology fosters trust, increases adoption, and enables the inclusion of rare or clinically obscure diseases that may otherwise be overlooked. Ultimately, this approach enhances the ontology's utility for precision medicine, rare disease diagnostics, and biomedical research, ensuring it remains adaptable and relevant to evolving needs.

## The Mondo resource

**Fig. 2.**
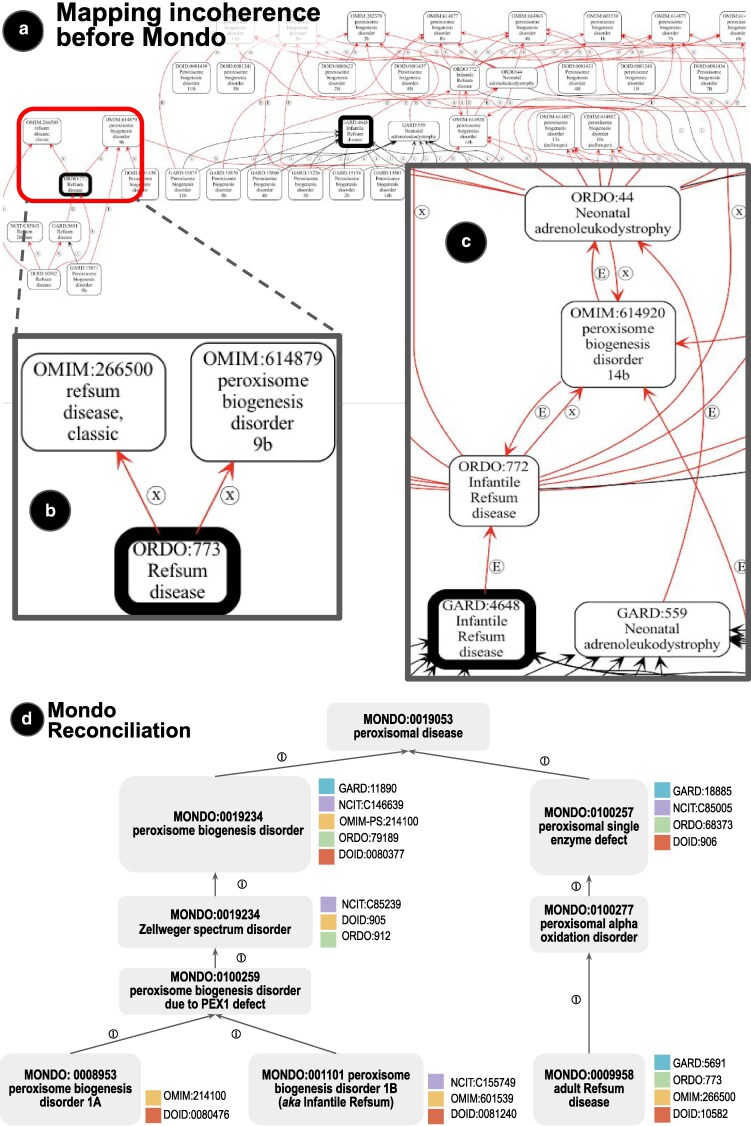
Mapping incoherence and reconciliation with Mondo. a) Global mapping incoherence. Visualization of walking all mappings on GARD, OMIM, Orphanet, NCIt, and DOID, starting from Refsum disease. Individual semantic imprecision accumulates, resulting in conflation of different concepts, such as (adult) Refsum, infantile Refsum, Zellweger, neonatal adrenoleukodystrophy, and gene-specific types. [(x) = xref (cross-reference), (E) = SKOS mapping]. b) Zoomed-in area showing imprecise mapping around Refsum. c) Zoomed-in area around infantile Refsum. d) Mondo reconciliation of a part of the peroxisomal disorders hierarchy. Previous confusion and conflation are resolved by a clear hierarchy, separating Zellweger spectrum disorders (left side) from nonbiogenesis disorders, such as (adult) Refsum disease. Infantile Refsum is resolved as a synonym of type 1B peroxisome biogenesis disorder. Mondo mappings are precisely resolved at the relevant level of specificity. Disease entities form the lowest level, with groupings above that. Note that for simplicity, not all subclasses are shown.

To reconcile the differences among disease terminology resources (Fig. 2), Mondo was created by the Monarch Initiative as an open, community-driven resource.

Designed with a focus on community collaboration, we ensure that Mondo remains comprehensive, accurate, and adaptable to the evolving needs of biomedical research and clinical communities. By engaging experts from various fields—such as rare disease specialists, clinical geneticists, and bio-ontologists—Mondo harmonizes disease terminologies and key attributes from major resources (Supplementary Table 1 and https://mondo.monarchinitiative.org/pages/sources/), including OMIM (Amberger and Hamosh 2017), Orphanet (Slebodnik 2009), DO (Schriml et al. 2022), and the National Cancer Institute Thesaurus (NCIt) (de Coronado et al. 2023). This collaborative model enables rapid updates through user-driven curation workflows and regular workshops, allowing new perspectives and clinical guidelines to be swiftly incorporated.

**Fig. 3.**
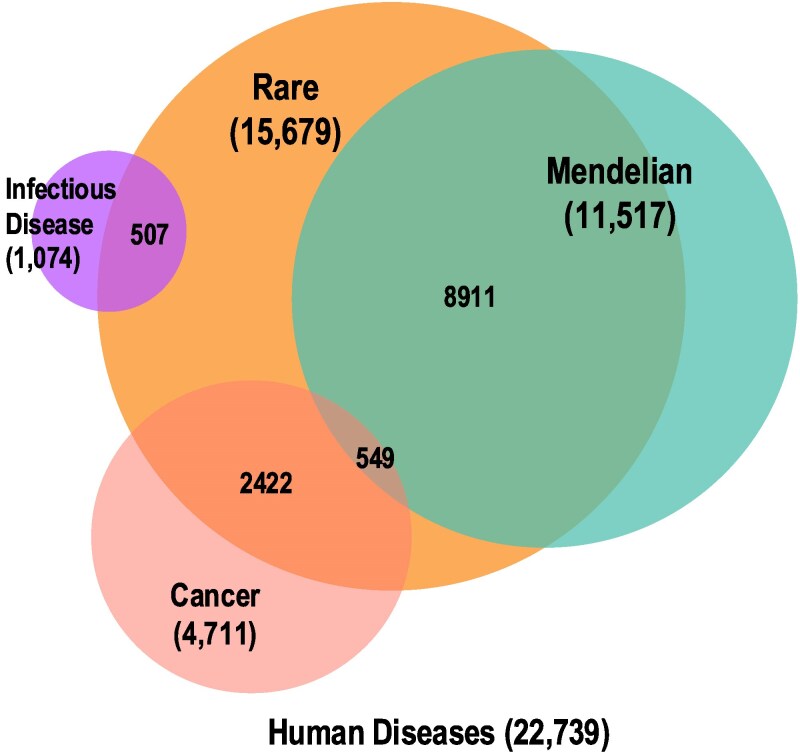
Representation of key disease types. Total number of human disease terms (descendants of “disease” (MONDO:0000001)) and overlap between the key human disease types; nonhuman diseases and susceptibilities are not included in these numbers. Note, there are an additional six nonrare Mendelian cancers that are not shown.

Mondo is a logic-based ontology that reconciles 21 disease resources (Supplementary Table 1), collectively representing approximately 100,000 source concepts, into >25,000 distinct disease concepts for both humans (>22,000) and nonhuman animals (∼3,000) (Table 1). The resources incorporated into Mondo were selected for their scope, strengths, and usage. The ontology covers various disease categories, such as rare genetic diseases (including ultra-rare n-of-1 diseases that are known to affect only one person), infectious diseases, cancers, and Mendelian diseases (Fig. 3). It also includes environmentally influenced diseases and complex genetic diseases that may not be documented in other sources. Mondo is developed in accordance with the principles of the Open Biological and Biomedical Ontology (OBO) Foundry (Jackson et al. 2021); each concept is assigned a permanent, unique identifier within the MONDO namespace. Mondo includes >110,000 synonyms—categorized as exact, narrow, broad, or related, using Simple Knowledge Organization System (SKOS) predicates (Supplementary Table 2).

**Table 1.** Disease terminologies and resources integrated in Mondo.

Disease resources (sorted alphabetically)	Number of mappings	Number of exact synonyms
Clinical Genome Resource (ClinGen)	N/A	1,865^a,b^
OMIM	8,841	10,605
Orphanet	9,195	13,227
National Cancer Institute Thesaurus (NCIt, limited to human and nonhuman neoplasms)	3,688	20,233
MedGen	21,272^a^	N/A
Genetic and Rare Diseases Information Center (GARD)	10,730^a^	N/A
International Classification of Diseases, 10th Revision (ICD 10)	1,121	540
International Classification of Diseases, 11th Revision—foundational component (ICD 11)	4,104	2,604
SNOMED CT	8,854	N/A
Nanbyo Disease Ontology (NANDO)	2,345^a,c^	N/A
Unified Medical Language System (UMLS)	21,272^a^	N/A
National Organization for Rare Disorders (NORD)	427^a^ (links to reports)	439^a,b^
Open Targets (OTAR, EFO)	2,400^a^	N/A
Disease Ontology (DO)	11,442	18,083
Online Mendelian Inheritance in Animals (OMIA)	2,648	168

Mondo version used is v2025-04-01. Mappings correspond to the number of exact mappings from the Mondo “disease” branch to a resource. The number of exact synonyms is the total number of synonyms linked to Mondo classes.

^a^Indicates content that is maintained by groups part of the biomedical community, including governmental organizations.

^b^Indicates that only the preferred labels are integrated into Mondo.

^c^Indicates that these mappings are not considered exact mappings.

### Mondo classification approach

Mondo provides a hierarchical structure that can be used to annotate data at varying levels of precision. The classification of disease concepts in Mondo is pluralistic, incorporating multiple perspectives based on clinical, biological, and literature contexts. For instance, geneticists may favor a gene-centric classification, while clinicians may prioritize a phenotypic-based approach. To accommodate these diverse viewpoints, Mondo allows multiple-parent classification of diseases, ensuring it serves a broad range of users. For example, “diabetes mellitus” (MONDO:0005015) is both an “endocrine pancreas disorder” (MONDO:0001933) and a “glucose metabolism disease” (MONDO:0002908). Specific diseases, such as monogenic disorders, are differentiated by gene-specific subtypes even if their phenotypic presentation is similar, for example, in the case of developmental and epileptic encephalopathies. These subtypes are represented as individual terms grouped under a more generic concept. For example, the general concept “developmental and epileptic encephalopathy” (MONDO:0100062) groups over 100 subtypes that represent the specific monogenic disease entities, such as “developmental and epileptic encephalopathy, 1” (MONDO:0010632), which is caused by a variation in the *ARX* gene.

### Mondo leverages the classifications of specialized reference ontologies

Mondo is deeply integrated with other ontologies. For example, Uber-anatomy ontology (Uberon) (Haendel et al. 2014) terms are used to relate diseases to the anatomical system affected; National Center for Biotechnology Information (NCBI) Taxonomy terms are used to relate infectious diseases to the causal infectious agent (and agent of transmission, where appropriate), as well as defining the taxa of nonhuman animal specific diseases; gene ontology (GO) terms are used to relate diseases to the affected and causal biological processes, etc. (Supplementary Table 3).

Reusing terms from other ontologies and importing their classifications offers various advantages. Firstly, we ensure that Mondo is interoperable with other widely used reference ontologies (such as Uberon and GO). Secondly, data related to other types of biomedical entities, such as anatomy or biological processes, are directly connected to Mondo classes, which help with data integration. Lastly, the classification of reference ontologies can be leveraged directly to inform Mondo classification. For example, the Mondo term “histoplasmosis meningitis” (MONDO:0001471) is defined as a “meningitis caused by the infectious agent ‘Histoplasma capsulatum’ (NCBITaxon:5037)”. Since “Histoplasma capsulatum” is classed within “fungi” (NCBITaxon:4751) according to NCBI Taxonomy, “histoplasmosis meningitis” is auto-classified as a “fungal meningitis” (MONDO:0006764) and “fungal infectious disease” (MONDO:0002041).

A considerable challenge in maintaining large ontologies like Mondo is the consistent description of related classes (eg classes which are conceptually similar, and thus share properties). Mondo uses the “Dead Simple OWL Design Pattern” (DOSDP) (Osumi-Sutherland et al. 2017) system to define templates for describing related diseases. These templates define how each disease type is represented in the ontology, including its preferred label and logical and textual definitions, ensuring a consistent representation. They serve as both curation guidelines/documentation (how should infectious disease be defined?) and a computational framework for automatically generating ontology axioms and annotations (such as labels or synonyms). While DOSDP templates are typically used to generate ontology classes from scratch (Stefancsik et al. 2023), the Mondo quality control (QC) system uses them primarily to ensure that manually curated disease classes (ie classes curated using Protege (Musen and the Protégé Team 2015) or ROBOT templates (Jackson et al. 2019)) are represented according to our curation guides. For example, by documenting that all monogenic diseases must have an association with a causal gene represented using a specifically formatted logical axiom in a DOSDP design pattern, we can ensure, during automated quality control checking, that this has been done correctly.

Mondo has over 100 disease templates to define the representation of terms based on species (eg human disease vs nonhuman disease), etiology (eg infectious disease, genetic disease), and anatomical system affected by the disease (eg cardiovascular disorder). All templates can be found on GitHub: https://github.com/monarch-initiative/mondo/tree/master/src/patterns/dosdp-patterns.

### Curated mappings

Articulating similarities between entities across a set of ontologies or terminologies that cover the same domain is most frequently done using mappings. These are represented in Mondo using the “database cross reference” (“xref”) property, and additionally supplied as mapping files following the Simple Standard for Sharing Ontology Mappings (SSSOM) standard (Matentzoglu et al. 2022a). Often, mappings link one term to a broader or narrower term if there is no exact correspondence (Kalfoglou and Schorlemmer 2003; Bellahsene et al. 2011). Mondo provides curated semantic mappings between source ontologies and terminologies, such as OMIM, International Classification of Diseases, 10th Revision, Clinical Modification (ICD10-CM), Orphanet, and the NCIt. Mondo uses the Bioregistry (Hoyt et al. 2022) to standardize the usages of prefixes, CURIEs, and URIs in such mappings, thereby promoting interoperability.

Additionally, precise mappings, database cross-references (dbxrefs), and linkouts to related resources are included in Mondo, and separately published in standardized formats such as SSSOM (Matentzoglu et al. 2022a). The Biomappings project (Hoyt et al. 2023) contributed nearly 200 exact mappings to Medical Subject Headings (MeSH).

### Releases

All release artifacts are preserved in the project's GitHub repository (https://github.com/monarch-initiative/mondo). We make standard and stable versions available for public download. GitHub records metadata such as dates when changes were made, who made the changes, and commit messages from developers. We also include documentation for users and software developers to facilitate the reuse of the ontology (https://mondo.monarchinitiative.org/pages/editors/). We encourage community members who modify or add to the Mondo code to submit their modifications via GitHub as Pull Requests to be considered for inclusion into our overall codebase.

We make monthly versioned releases of Mondo. All major releases (ontology, mappings, source data) are fully documented and have stable, persistently resolvable URLs for reference in publications. Each ontology version includes instructions for replacing obsolete terms.

### Provenance and attribution

A major downside of many integrative efforts is the lack of appropriately documented information provenance. We aim to make disease information, including all mappings, Findable, Accessible, Interoperable, and Reusable (FAIR) within Mondo (Wilkinson et al. 2016). All information in Mondo, including synonyms, definitions, SubClassOf axioms, is supported by provenance. This provenance information includes references to the disease terminologies (eg NCIt, Orphanet, etc) or resources (eg MedGen, NORD, etc) where the information came from, including publications, and/or personal communication with experts or expert groups (eg ClinGen). For example, the term “alpha thalassemia spectrum” (MONDO:0011399) includes the exact synonym “alpha-thalassemia”, as supported by OMIM (OMIM:604131), DO (DOID:1099), and Orphanet (Orphanet:846); this provenance is recorded as a database cross-reference (xref) on the synonym in Mondo. The classification of the disease as rare is reflected by its inclusion in the Orphanet and Genetic and Rare Diseases Information Center (GARD) rare disease subsets. In addition to their provenance, information is attributed to the group or individuals who created (or contributed to) the information. For example, the cross-reference of “alpha thalassemia spectrum” to Unified Medical Language System (UMLS) (UMLS:C0002312) was created by the MedGen team as indicated by the source annotation “MONDO:MEDGEN”.

### Alignment with external disease resources

Mondo integrates disease resources that evolve constantly, which makes it crucial to have a system that keeps Mondo in alignment. To address this, the overall Mondo curation workflow includes a semiautomated synchronization workflow to import terms represented in these resources. First, the latest version of a resource is downloaded and processed to follow Mondo metadata standards. Next, a lexical matching tool (Ontology Access Kit, OAK lexmatch (Mungall et al. 2025)) is used to determine if any new terms (not yet mapped to Mondo) have a possible match that is already included in Mondo. If so, that mapping is reviewed by a curator and added to Mondo. If no such mapping can be found (also verified by a curator), the term is integrated into Mondo as a new term along with its classification. As part of the integration process, we import term labels, synonyms, human-readable definitions, obsoletion status, and classification (in particular, the parents under which a disease should be classified). Apart from mapping and classification decisions for new terms, all parts of the synchronization workflow are fully automated. This enables near real-time synchronization with key disease resources such as OMIM and Orphanet. The Mondo synchronization pipeline is open source and available on GitHub (https://github.com/monarch-initiative/mondo-ingest). The current alignment system runs once per month and has a runtime of about 5 h, which is mainly due to processing large sources such as NCIT. Most of that time goes towards automated matching processes. The pipeline is constantly optimized to handle the ever-growing external resources and any resulting scalability issues. More details about how records from external disease resources are represented in Mondo and how complexities of each resources are handled in Mondo can be found in our documentation (eg OMIM records representation in Mondo can be found here: https://mondo.readthedocs.io/en/latest/editors-guide/OMIM-representation).

### Representation of newly identified diseases

Mondo aims to provide a comprehensive representation of all diseases, including newly identified conditions and extremely rare syndromes that may not yet be represented in other disease resources. We rely on the expertise of collaborators and community members working at the forefront of medicine, diagnostics, and patient engagement to identify the need for new Mondo terms. New disease concepts are included in Mondo when supported by evidence, such as a peer-reviewed publication or clinical consensus. This evidence is recorded with the term to ensure transparency and traceability. Term requests are managed through our open repository, and a list of newly created terms is published with each release, providing both transparency and an opportunity for community review and challenge.

### Quality control

Mondo is maintained via a diverse set of automated and semi-automated workflows (including the source alignment workflow described above) (https://mondo.readthedocs.io), as well as manually, based on user and expert requests submitted on GitHub. With more than 70 pull requests per month on average (3,818 opened pull requests over the last 51 months), an extensive array of QC checks and continuous integration tests is needed, both at the time of curation (triggered via GitHub Actions when a pull request, eg an edit request, is submitted) and as part of the release process. These QC checks report the occurrence of errors such as invalid classifications, duplicate synonyms or more specific problems with the logical structure. For example, there is a QC check reporting whether a genetic disease is a child of another genetic disease related to a different gene [which would constitute an error according to Web Ontology Language (OWL) ontology semantics]. Many of the tests developed for Mondo have been standardized and shared with the wider ontology community through our support of the Ontology Development Kit (ODK) (Matentzoglu et al. 2022b). Such automation is essential to ensure the usability, scalability, and relevance of the Mondo resource far into the future, thereby attracting greater uptake and investment by the biomedical community.

## Mondo's community-centric approach

The strength and utility of Mondo lie in the community that helps build and sustain it. Mondo's development is community-driven, with current active involvement from over 100 clinical experts and domain specialists from over 25 institutions, including OMIM (“OMIM” n.d.), Orphanet (“Orphanet” n.d.), Clinical Genome Resource (ClinGen) (Clinical Genome Resource n.d.), GARD (“Genetic and Rare Diseases Information Center” n.d.), National Organization for Rare Disorders (NORD) (“National Organization for Rare Disorders” 2022), and Nanbyo data (“NanbyoData” n.d., “DBCLS,” n.d.) (see full list here: https://mondo.monarchinitiative.org/pages/contributors/), who work together to continuously evolve and improve the resource.

### Consensus building

Differences of opinion and ambiguities inevitably arise when integrating and harmonizing disease concepts, and Mondo addresses these through an inclusive and transparent resolution process. Wherever possible, multiple classifications and alternative names are preserved, and all provenance is captured so that users can make informed decisions based on their needs. Best practices and decision-making rules are documented to ensure clarity and reproducibility. When divergent views cannot be equally accommodated—for example, when deciding whether or not a term represents a true disease entity—Mondo relies on domain expertise to establish consensus. This involves consulting the literature, engaging subject matter experts across multiple domains, and gathering input from medical professionals, clinical researchers, and/or patient advocacy groups, such as through the Epilepsy Workshop, held over three sessions in 2024–2025 (Vasilevsky et al. 2025, described in more detail below). All discussions are documented on the public GitHub tracker, where they are open for community review and comment. For areas of active debate, we convene focused community meetings or workshops to support in-depth discussion and resolution. All decisions, along with supporting evidence and provenance, are communicated to the community for feedback before implementation. Importantly, ontology development is inherently iterative: as new data and perspectives emerge, decisions are revisited to ensure that Mondo remains a dynamic, evidence-based resource that adapts to evolving scientific knowledge.

### Mondo workshops with domain experts

When reviewing specific clinical domains, Mondo aims to engage expert groups (eg ClinGen expert panels, rare disease foundations, etc.) to ensure that Mondo is correct, relevant, reflects current clinical views, and addresses community needs. Expert engagement is also done through virtual or in-person workshops, which bring together varied perspectives to help inform Mondo's decisions regarding ontology updates.

The inaugural Mondo workshop (“Workshop” 2025) was held in November 2018 with over 40 curators, ontology developers, and clinicians in attendance, from ontologies including the Human Phenotype Ontology (HPO), the Experimental Factor Ontology (EFO), and NCIt, and curation groups including ClinGen, EMBL's European Bioinformatics Institute (EMBL-EBI), Orphanet, GARD, MedGen, and OMIM. The goal of the inaugural workshop was to exchange curation processes and challenges, and determine requirements for how Mondo could assist in disease definitions across the community.

Since then, we have hosted eight more workshops aimed at gathering experts’ input on disease representation and Mondo ontology improvements. The latest Mondo-hosted workshop in late 2024/early 2025 was a three-part virtual workshop focused on epilepsy, which brought together clinicians and geneticists with specialities in epilepsy, as well as representatives from disease terminologies, and patient advocacy groups (Vasilevsky et al. 2025). The workshop also resulted in new broader disease categories and/or concept suggestions including immune epilepsy, genetic epilepsy, generalized epilepsy, and epilepsy syndrome. All workshops hosted by Mondo are listed on our website: https://mondo.monarchinitiative.org/pages/workshop/.

### Mondo outreach seminars

Mondo hosts monthly outreach seminars (“Mondo Outreach Call”) that bring together stakeholders and clinical terminology experts to share insights, discuss specific use cases, and help guide the ongoing development of Mondo (https://mondo.monarchinitiative.org/pages/workshop/#outreach). Each call features updates from the Mondo curation team, followed by a presentation and open discussion with invited users or groups. These presentations often highlight work related to Mondo and provide valuable context for how Mondo can better support the broader community. The goals of this ongoing series are to foster collaboration, gather feedback, and ensure that Mondo continues to evolve in alignment with user needs. Between June 2023 and May 2025, the Mondo team hosted 20 outreach sessions for data analysts, data integrators and other users of Mondo.

### Community curation

Community users request new terms, suggest revisions (such as new disease classifications), and report issues (such as misclassifications) via issues/tickets on the Mondo GitHub issue tracker. Any Mondo user can create a free GitHub account and contribute to discussions, decisions, and proposed changes in the ontology. With more than 5,092 pull requests and 3,932 issues submitted since its inception in 2017, Mondo has seen a 2.3-fold increase in community engagement based on the number of unique contributors on GitHub (Supplementary Fig. 1). The GitHub issue tracker ensures transparency and documentation of all discussions, decisions, and changes. The Mondo curation team addresses these issues, often in collaboration with our users.

**Fig. 4.**
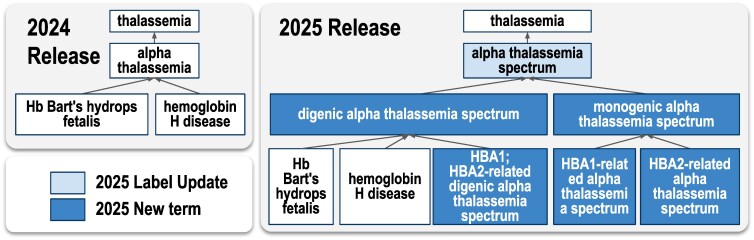
Updates to alpha thalassemia classes. The 2024 release shows the prior architecture of the alpha thalassemia branch, which contained only two child terms. Engagement with a ClinGen expert group resulted in a more robust and clinically accurate representation (2025) of alpha thalassemia.

For example, engagement with a ClinGen expert group resulted in a more robust and clinically accurate representation of the alpha-thalassemia branch of Mondo, specifically for use in gene curation by the ClinGen General Gene Curation Expert Panel (General GCEP) and the ClinGen Hemoglobinopathy Variant Curation Expert Panel (VCEP). During this engagement, ClinGen, along with other community partners (eg OMIM, MedGen), was able to collaborate on the proposed restructuring of the ontology for community consensus (see Fig. 4) (https://github.com/monarch-initiative/mondo/issues/7561).

To support the growing scale of community involvement, we introduced the use of Knowledge Graph Change Language (KGCL) requests, allowing contributors to more easily propose and track changes (Hegde et al. 2025). KGCL comes with a natural language interface that allows users to simply add or modify content through GitHub issue comments without knowing anything about the underlying representation of Mondo. For example a user might request to “change synonym “ZCCHC8-related telomere biology disorder” for MONDO:0032865 to “ZCCHC8-related telomere biology disorder” without the need to know specialized ontology curation software such as Protégé. This approach has helped streamline community curation and foster greater collaboration. Recently, agentic AI has reached a level of maturity that allows for even more complex forms of automation. Mondo is now routinely using readily available AI systems such as Claude, Copilot, or Dragon-AI (Toro et al. 2024) to analyze issues, propose solutions, and even edit the ontology directly. Human curators act sometimes as reviewers, sometimes as authors whose work is reviewed by an AI agent—but they are always in the loop.

To facilitate inclusive user engagement, we have developed tools such as ontobot (Hegde et al. 2025) that can automatically add content to the ontology following comments on the Mondo issue tracker.

### Community-managed content

Curating and keeping disease terminologies current is costly. With new diseases, especially disease subtypes, being defined and discovered every month, open data and ontology projects like Mondo rarely have the resources to handle the scale of the curation required. We address this issue by closely involving and training key community partners (such as NORD and MedGen) to contribute well-defined, expertly curated knowledge directly to Mondo. Some Mondo community groups expertly curate disease information in their own projects, including mappings, synonyms, gene associations, rare disease designations, and language translations (currently, Japanese). We have developed a curation procedure that enables those groups to directly contribute and keep current such content in spreadsheets, which are imported automatically into Mondo. All such community-managed content comes with extensive provenance and attribution metadata. For example, “amyloidosis cutis dyschromia” (MONDO:0017906) was designated as a rare disease by NORD, which is attributed by a source annotation such as MONDO:NORD, and the mappings to MedGen (MEDGEN:1641859) and UMLS (UMLS:C4554601) were curated by the MedGen team (source annotation “MONDO:MEDGEN”). As of the time of this writing, five kinds of community-provided content are integrated into Mondo: cross-references (from GARD, NORD, MedGen, DBCLS/NANDO, Open Targets/EFO), synonyms (to record disease naming preferences, from ClinGen and NORD), linkouts (from ClinGen and Malacards), and various tags, including rare designations from NORD, GARD, and Orphanet and usage tracker from ClinGen and Open Targets. Capturing this information is useful for multiple reasons, such as generating community-specific subsets (see Customization) of Mondo, and keeping a record of the expert groups using this term that could be consulted when major changes to the term are necessary (eg obsoletion, re-classification).

### Accessing Mondo

To meet the needs of a diverse and growing community, we provide multiple ways to interface with the Mondo ontology. The Monarch Initiative website (https://monarchinitiative.org/) includes an intuitive search tool to look up disease terms by ID or by synonymous names, and returns useful information about associated phenotypes, and causally linked genes. Independently, EMBL-EBI distributes Mondo via their Ontology Lookup Service (OLS) browser (https://www.ebi.ac.uk/ols4/ontologies/mondo) where users can interactively explore hierarchically-organized terms. Note that the official Monarch Initiative web pages are connected to a large knowledge graph and thus may be refreshed less frequently than Mondo—if a user wishes to browse the very latest Mondo version they should use OLS, which updates its ontologies every night.

Alternatively, for users who wish to perform more high-throughput analyses using the Mondo ontology, we also distribute Mondo as OWL- and JSON-formatted files for download (at https://mondo.monarchinitiative.org/pages/download/ or https://github.com/monarch-initiative/mondo/releases). Several packages developed by our team enable highly efficient and customizable queries on Mondo, including *monarch-py* for Python users (https://github.com/monarch-initiative/monarch-app/tree/main/backend) and *monarchr* for R users (https://github.com/monarch-initiative/monarchr). These packages abstract away much of the complexity of working with large ontological resources like Mondo and integrating them with additional resources, such as gene annotations and cross-ontology ID mappings.

## Disease harmonization, nomenclature, and curation practices for a wide community

### Disease naming

One challenge in integrating disease terminologies is the lack of consensus between different experts on naming, definition, and classification. For example, clinicians often focus more on phenotypic features, while geneticists prefer gene-based naming and patient groups more often use everyday language to refer to diseases. Mondo's goal is to accommodate a wide range of use cases and perspectives (Thaxton et al. 2024) with both rigor and transparency.

Disease names also evolve due to cultural, biological, and patient-driven factors. As understanding of a disease deepens, names may change to reflect new scientific knowledge as in cases like “Lou Gehrig disease” (now amyotrophic lateral sclerosis (MONDO:0004976)), or address cultural concerns, such as “Wegener syndrome” (now granulomatosis with polyangiitis (MONDO:0012105)).

Mondo aims to reflect all community preferences for disease naming without arbitrating official names. For each disease, we provide a list of synonyms and track their provenance. We also allow community-specific preferred names, such as “ERF-related craniosynostosis” (MONDO:0010929), which is the dyadic name preferred by ClinGen. Additional exact synonyms for this term include “craniosynostosis 4”, “craniosynostosis caused by mutation in ERF”, “craniosynostosis type 4” and “ERF craniosynostosis”. This approach ensures Mondo serves a wide range of communities.

### Customization

As described above, we have a system in place for adopters to indicate the preferred names and synonyms for a disease concept. We can provide customized Mondo subsets using an organization's preferred names, while maintaining a consistent ontology. For example, a specialized version of Mondo is provided for ClinGen, which preserves the full logical structure and metadata for all diseases, but excludes nonhuman animal diseases and replaces Mondo's default preferred labels with those preferred by ClinGen. In addition, we are leveraging standard GitHub webhooks to directly communicate release changes to ClinGen's curation system, which enables curators to directly respond to changes in Mondo, such as term merges or name changes.

We are now offering language translations, with a Japanese version of Mondo now available. This system functions similarly to the language translation model, which the Monarch Initiative team has already pioneered for the HPO (Gargano et al. 2024).

### Disease lumping or splitting

The need to lump and split disease entities has been recognized as the key challenge of nosology for over five decades (McKusick 1969; Biesecker et al. 2021; Hamosh et al. 2021). Lumpers group diseases into broader categories that include concepts that share important features despite some differences; splitters prefer narrower disease categories that subgroup disease entities. ClinGen has developed frameworks to determine whether diseases should be “lumped” or “split” (Thaxton et al. 2022). However, many philosophies and approaches exist, and disease sources (often guided by their individual use cases) often disagree on these disease lumps or splits. In addition, whether a term A is split into terms B and C, versus terms B and C are lumped together into term A, is a question of perspective.

**Fig. 5.**
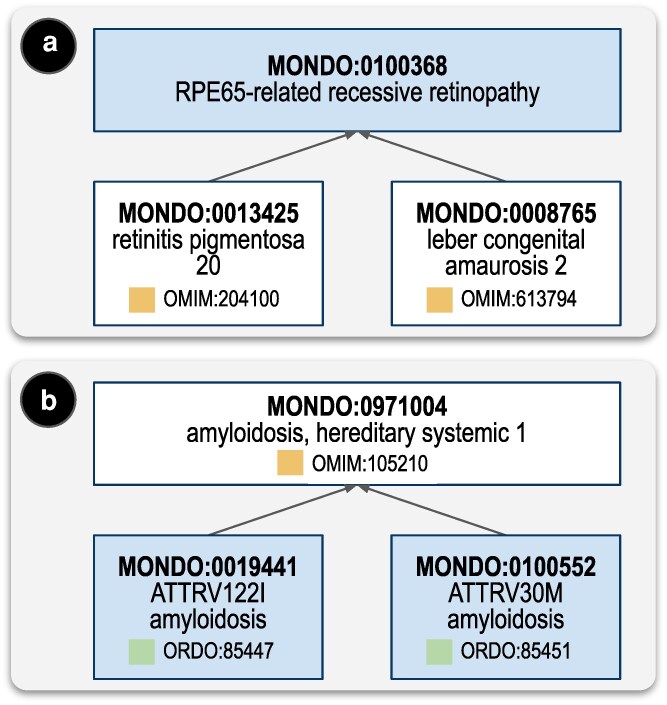
Lumping and splitting (a) the two separate disease entities recognized by OMIM, “retinitis pigmentosa 20” (OMIM:613794; MONDO:0013425) and “leber congenital amaurosis 2” (OMIM:204100; MONDO:0008765), are “lumped” into the disease entity, “RPE65-related recessive retinopathy” (MONDO:0100368), represented as the ontological parent (ie superclass) of “retinitis pigmentosa 20” and “leber congenital amaurosis 2” (b) The term “amyloidosis, hereditary systemic 1” (MONDO:0971004, OMIM:105210), considered in OMIM as a single disease entity, is “split” into “ATTRV122I amyloidosis” (MONDO:0019441, Orphanet:85451) and “ATTRV30 M amyloidosis” (MONDO:0100552, Orphanet:85447). These “split” terms are represented in Mondo as ontological children (ie subclasses) of “amyloidosis, hereditary systemic 1”.

In the spirit of serving the community as a whole, we do not arbitrate whether a disease entity should be split or lumped; rather, we represent all views in Mondo, either by creating new terms (Fig. 5a), or by creating the appropriate relations between existing terms (Fig. 5b).

### Nonhuman animals

Mondo covers a broad range of diseases in nonhuman animals, addressing use cases from the veterinary domain and model organisms. Animal diseases are classified separately from human diseases as subtypes of “nonhuman animal disease” (MONDO:0005583) for users who wish to focus strictly on human diseases. However, Mondo connects analogous nonhuman animal and human diseases (eg “acromegaly, domestic cat” (MONDO:1010563) is a cross-species analog of “acromegaly” (MONDO:0019933)) to facilitate cross-species disease comparisons and appropriate experimental model selection. These relations are also leveraged for automated nonhuman animal disease classification, which mirrors the classification of human diseases. Mondo includes diseases from Online Mendelian Inheritance in Animals (OMIA) (Nicholas and Tammen 1995), which are mainly genetic and hereditary. Additional nonhuman animal diseases include neoplasias (eg from NCIt) and infectious diseases.

### Relationship between Mondo and HPO

Mondo is co-developed with the HPO to ensure alignment and interoperability, creating a more holistic semantic resource for diseases. A subset of HPO classes is imported into Mondo and used in logical disease definitions; for example, MONDO:0003337 “acute hemorrhagic encephalitis” is defined as encephalitis with the feature “abnormal bleeding” (HP:0001892). In this framework, Mondo and HPO distinguish between disease entities and phenotypic features. A disease entity has an etiology, one or more phenotypic features—often with varied onset—a characteristic time course, and sometimes a typical treatment response. In contrast, phenotypic features are atomic observations, usually independent of time course, and can occur across many diseases with different causes (eg tremor). Diseases represent constellations of such features, while phenotypes are their components; however, there is a gray zone where a condition may function as both, such as diabetes mellitus (MONDO:0005015), which is a disease but also a feature of syndromes like Bardet-Biedl (MONDO:0015229). To disambiguate, Mondo sometimes applies a unique label (eg “(disease)”) to distinguish a disease entity from the corresponding HPO phenotype term.

## Global adoption

Mondo is built with a community-first mindset and is widely used in global health initiatives and research projects. The Monarch Initiative uses Mondo's equivalence mappings to simultaneously search genomic resources through a unified disease interface (Putman et al. 2023). ClinGen uses Mondo as a foundational ontology to support disease curation efforts by providing stable identifiers, synonym tracking, and structured disease naming guidance that aligns with ClinGen's dyadic naming framework for monogenic diseases (Thaxton et al. 2024). Beyond rare diseases, Mondo's hierarchical structure facilitates investigation of complex disease etiologies through multiscale data integration. The ontology's explicit modeling of disease subtypes (eg separating monogenic Parkinson's disease forms from idiopathic forms) enables genome-wide association studies (GWAS) meta-analyses that distinguish shared versus distinct genetic architectures (Dulski et al. 2022).

### Adoption and community

Mondo is used across a diverse set of organizations to standardize disease terminology and improve data interoperability in research, clinical care, and drug development. For example, ClinGen and MedGen use Mondo as a central authority for defining and curating disease entities, while platforms like Kids First and C-Path's RDCA-DAP use it to harmonize disease data across large-scale genomic and clinical datasets. Organizations like Every Cure and IMO Health (see Table 2) leverage Mondo to enable artificial intelligence (AI)-driven drug repurposing and enhance the utility of electronic health records. OMIM web service output includes a field for Mondo identifiers (see https://omim.org/help/api). MedGen provides prominent linkouts to Mondo on their website (eg https://www.ncbi.nlm.nih.gov/medgen/44287). International collaborations, such as with Database Center for Life Science in Japan (DBCLS) and European Molecular Biology Laboratory's European Bioinformatics Institute in the UK (EMBL-EBI), support global integration and translation of Mondo, ensuring its role as a foundational resource for consistent disease representation. We are in active discussion with the World Health Organization (WHO) on integrating Mondo more closely with ICD-11. In addition to being used widely in Europe, United States, and Japan, Mondo is used in China (Wu et al. 2021; Lu et al. 2023), Australia (Tudini et al. 2022), Canada (eg https://docs.cqdg.ca/docs/fonctionalit%C3%A9s), and through global organizations such as GH4GH (Wagner et al. 2021). Mondo's impact on these resources and organizations can be partially quantified by looking at how many terms they have linked content to. ClinGen, for example, is currently using 1,297 Mondo terms to standardize disease terms and identifiers across its gene–disease validity curation efforts; Open Targets, a major effort for therapeutic drug target identification, currently links its content to 9,815 diseases in Mondo; NORD presents information to patients on their web pages that currently includes 10,117 rare diseases in Mondo. To increase our reach, we have been running an outreach seminar series (see Mondo Outreach Seminars) where current and prospective groups are invited to present their use case for Mondo. A breakdown of all current adopters and contributors of Mondo can be found on our website (https://mondo.monarchinitiative.org/pages/users/). A subset of current Mondo adopters and end users is highlighted in Table 2.

**Table 2.** Subset of current Mondo adopters and end users.

Organization	Website	How Mondo is used
ClinGen	https://clinicalgenome.org/	Mondo is the central coding authority for disease terms, supporting data consistency and interoperability across ClinGen's expert curation tools, public applications, website display, and internal streaming services.
Critical Path Institute (C-Path)	https://c-path.org/	Mondo is used to standardize and integrate disease information in rare disease datasets within the RDCA-DAP platform.
Database Center for Life Science (DBCLS)	https://dbcls.rois.ac.jp/index-en.html	Supports Mondo through mapping to the Nanbyo Disease Ontology and Japanese translation; enables its use in standardizing patient data in Japanese cohort studies.
EMBL's European Bioinformatics Institute (EMBL-EBI)	https://www.ebi.ac.uk/	Uses Mondo as the source of disease concepts within the experimental factor ontology (EFO), supporting projects like Open Targets and the GWAS Catalog.
Every Cure	https://everycure.org/	Uses Mondo-derived disease list in its Matrix platform to identify drug repurposing candidates using AI and knowledge graphs.
IMO Health	https://www.imohealth.com/	Integrates Mondo into EHR platforms to enable secondary use of clinical data, discover rare disease phenotypes, and bridge clinical documentation with research terminology.
INCLUDE	https://includedcc.org/	Standardizes and harmonizes disease information in Down syndrome datasets using Mondo, enabling virtual cohort building and cross-study comparisons.
Kids First Data Resource Portal	https://portal.kidsfirstdrc.org/	Standardizes and harmonizes disease information in pediatric datasets using Mondo, enabling virtual cohort building and cross-study comparisons.
MedGen	https://www.ncbi.nlm.nih.gov/medgen/	An NCBI, NLM-run project that uses Mondo identifiers to define disease entities and relationships in ClinVar, GTR, and MedGen; manages UMLS mappings and provides feedback to the Mondo team to enhance content.
NORD	https://rarediseases.org/	Leverages Mondo for cross-references, associated gene and synonym data, disease hierarchy creation, and serves as a rare disease content authority.
OMIA	https://omia.org/	Integrates nonhuman animal disease data with Mondo.
PomBase	https://www.pombase.org/	Uses Mondo to annotate human disease orthologs in yeast and refine disease ontology based on conserved pathway feedback.

### Sustainability

For more than seven years, Mondo has been supported through various funding sources (see Funding section), but its long-term sustainability comes from being a true community-driven project, managed openly on GitHub. Regular contributions from organizations with major buy-in such as ClinGen, NORD, EBI, IMO, and Every Cure ensure that Mondo remains actively maintained even without dedicated curation time. Similar community-based efforts, like the Uberon anatomy ontology (Haendel et al. 2014), have thrived for over more than a decade with limited dedicated funding thanks to ongoing community engagement.

### Mondo as a FAIR vocabulary

OBO principles predate FAIR principles, and can be considered as a concrete, actionable implementation of FAIR. Mondo adheres strongly to the community standards set by the OBO principles (Jackson et al. 2021) (see OBO Dashboard: https://dashboard.obofoundry.org/dashboard/mondo/dashboard.html). It provides explicit licensing, versioning (GitHub and versioned releases) and makes heavy use of GUPRIs (globally unique and persistent identifiers, known in the OBO world as PURLs, persistent URLs). Xu et al. (2023) define a set of FAIR Vocabulary Features (FVFs) such as persistent identifiers, rich metadata, accessibility protocols, cross-references, versioning, licensing, and compliance with community standards. A basic self-assessment of Mondo against the FVFs mentioned in Xu et al. showed full compliance across all 11 features.

## Future plans

Over the years, the Mondo project has evolved through the integration of various technologies and methodologies. Early efforts focused on leveraging OWL reasoning and design patterns to structure and validate ontological data, while the use of more advanced tooling like Boomer (Mungall et al. 2016) enabled the incorporation of probabilistic reasoning to enhance quality control. These approaches helped automate many aspects of data validation, significantly improving efficiency and consistency.

We are also implementing Ontaccord, a Delphi-style platform for ontologies developed in our group (McMurry 2025), to gather community feedback and consensus on disease synonyms, definitions, and classifications. This workflow will effectively complement ongoing community engagement efforts in disease curation, enabling broader participation, and facilitating the formalization of consensus-driven decisions.

Integration with emerging AI/machine learning methods is a priority. We are designing novel human-in-the loop curation workflows where AI is employed to act as a junior curator to suggest content (which will then be reviewed by an expert curator) or as a reviewer (a human generates the original content). These curation workflows will come with an improved provenance model that clearly states if a statement was suggested by an AI system. More recently, we have explored the potential of generative AI through the DRAGON-AI evaluation, assessing how these technologies can support ontology development and refinement (Toro et al. 2024). This line of investigation sets the stage for future innovations. Looking ahead, we are poised to explore agentic AI-based workflows to further enhance automation and intelligent assistance in ontology management.

These efforts will further enhance Mondo's ability to support precision medicine applications, improving diagnostic accuracy and treatment outcomes.

## Conclusion

Mondo will continue to play a key role in integrating data and knowledge across multiple genomic and clinical research initiatives, further aligning and integrating with global standards supported by organizations such as the WHO, HL7, OHDSI, and GA4GH and by implementation in EHR Systems through recent partnerships with organizations such as IMO (McCormick n.d.). As new disease characteristics are revealed across disciplines, Mondo welcomes contributions and partnerships to ensure consistency and interoperability of disease definitions in a global community.

## Supplementary Material

iyaf215_Supplementary_Data

## Data Availability

Mondo is available at https://github.com/monarch-initiative/mondo. Supplemental material available at GENETICS online.

## References

[iyaf215-B1] Amberger JS, Hamosh A. 2017. Searching Online Mendelian Inheritance in Man (OMIM): a knowledgebase of human genes and genetic phenotypes. Curr Protoc Bioinformatics. 58:1.2.1–1.2.12. 10.1002/cpbi.27. PMC5662200 28654725

[iyaf215-B2] Bellahsene Z, Bonifati A, Duchateau F, Velegrakis Y. 2011. On evaluating schema matching and mapping. In: Bellahsene Z, Bonifati A, Rahm E, editors. Schema matching and mapping. Springer Berlin Heidelberg. p. 253–291.

[iyaf215-B3] Bello SM et al 2018. Disease ontology: improving and unifying disease annotations across species. Dis Model Mech. 11:dmm032839. 10.1242/dmm.032839. 29590633 PMC5897730

[iyaf215-B4] Biesecker LG et al 2021. Response to Hamosh et al. Am J Hum Genet. 108:1809–1810. 10.1016/j.ajhg.2021.07.006. 34478656 PMC8456172

[iyaf215-B5] Clinical Genome Resource . n.d. “Welcome to ClinGen.” [accessed 2025 April 18]. https://clinicalgenome.org/

[iyaf215-B6] “Database Center for Life Science | DBCLS” . n.d. [accessed 2025 April 28]. https://dbcls.rois.ac.jp/about-en.html

[iyaf215-B7] de Coronado S, Remennik L, Elkin PL. 2023. National Cancer Institute Thesaurus (NCIt). In: Health informatics. Springer International Publishing. p. 395–441.

[iyaf215-B8] Dulski J, Uitti RJ, Ross OA, Wszolek ZK. 2022. Genetic architecture of Parkinson's disease subtypes—review of the literature. Front Aging Neurosci. 14:1023574. 10.3389/fnagi.2022.1023574. 36337703 PMC9632166

[iyaf215-B9] Gargano MA et al 2024. The human phenotype ontology in 2024: phenotypes around the world. Nucleic Acids Res. 52:D1333–D1346. 10.1093/nar/gkad1005. 37953324 PMC10767975

[iyaf215-B10] “Genetic and Rare Diseases Information Center.” n.d. [accessed 2025 April 18]. https://rarediseases.info.nih.gov/

[iyaf215-B11] Haendel MA et al 2014. Unification of multi-species vertebrate anatomy ontologies for comparative biology in Uberon. J Biomed Semantics. 5:21. 10.1186/2041-1480-5-21. 25009735 PMC4089931

[iyaf215-B12] Haendel MA et al 2018. A census of disease ontologies. Annu Rev Biomed Data Sci. 1:305–331. 10.1146/annurev-biodatasci-080917-013459.

[iyaf215-B13] Hamosh A et al 2021. Response to Biesecker et al. Am J Hum Genet. 108:1807–1808. 10.1016/j.ajhg.2021.07.004. 34478655 PMC8456153

[iyaf215-B14] Hegde H et al 2025. A change language for ontologies and knowledge graphs. Database (Oxford). 2025:baae133. 10.1093/database/baae133. 39841813 PMC11753292

[iyaf215-B15] Hoyt CT et al 2022. Unifying the identification of biomedical entities with the bioregistry. Sci Data. 9:714. 10.1038/s41597-022-01807-3. 36402838 PMC9675740

[iyaf215-B16] Hoyt CT, Hoyt AL, Gyori BM. 2023. Prediction and curation of missing biomedical identifier mappings with biomappings. Bioinformatics (Oxford, England). 39:btad130. 10.1093/bioinformatics/btad130. 36916735 PMC10076045

[iyaf215-B17] Hügle B et al 2011. Hoffman syndrome: new patients, new insights. Am J Med Genet A. 155A:149–153. 10.1002/ajmg.a.33678. 21204224

[iyaf215-B18] “Human Disease Ontology Release v2022-03-02.” n.d. [accessed 2022 April 11]. http://purl.obolibrary.org/obo/doid/releases/2022-03-02/doid.owl

[iyaf215-B19] Jackson RC et al 2019. ROBOT: a tool for automating ontology workflows. BMC Bioinformatics. 20:407. 10.1186/s12859-019-3002-3. 31357927 PMC6664714

[iyaf215-B20] Jackson R et al 2021. OBO foundry in 2021: operationalizing open data principles to evaluate ontologies. Database (Oxford). 2021:baab069. 10.1093/database/baab069. 34697637 PMC8546234

[iyaf215-B21] Kalfoglou Y, Schorlemmer M. 2003. Ontology mapping: the state of the art. Knowl Eng Rev. 18:1–31. 10.1017/S0269888903000651.

[iyaf215-B22] Kamdar MR, Tudorache T, Musen MA. 2017. A systematic analysis of term reuse and term overlap across biomedical ontologies. Semant Web. 8:853–871. 10.3233/sw-160238. 28819351 PMC5555235

[iyaf215-B23] Lu M . 2023. TWAS Atlas: a curated knowledgebase of transcriptome-wide association studies. Nucleic Acids Res. 51:D1179–D1187. 10.1093/nar/gkac821. 36243959 PMC9825460

[iyaf215-B24] Mangaraj S, Sethy G. 2014. Hoffman's syndrome—a rare facet of hypothyroid myopathy. J Neurosci Rural Pract. 5:447–448. 10.4103/0976-3147.140025. 25288869 PMC4173264

[iyaf215-B25] Matentzoglu N et al 2022a. A simple standard for sharing ontological mappings (SSSOM). Database (Oxford). 2022:baac035. 10.1093/database/baac035. 35616100 PMC9216545

[iyaf215-B26] Matentzoglu N et al 2022b. Ontology development kit: a toolkit for building, maintaining and standardizing biomedical ontologies. Database (Oxford). 2022:baac087. 10.1093/database/baac087. 36208225 PMC9547537

[iyaf215-B27] McCormick P . n.d. Mondo 101: your guide to clinical terminology for rare diseases. IMO Health. [accessed 2025 May 5]. https://www.imohealth.com/resources/mondo-101-your-guide-to-clinical-terminology-for-rare-diseases/

[iyaf215-B28] McKusick VA . 1969. On lumpers and splitters, or the nosology of genetic disease. Perspect Biol Med. 12:298–312. 10.1353/pbm.1969.0039. 4304823

[iyaf215-B29] McMurry J . 2025. Ontaccord: an extensible platform for community consensus on ontological content. Zenodo. 10.5281/ZENODO.15103746.

[iyaf215-B30] Mungall C et al 2025. INCATools/ontology-Access-Kit: v0.6.22. Zenodo. 10.5281/ZENODO.15151634.

[iyaf215-B31] Mungall CJ, Koehler S, Robinson P, Holmes I, Haendel M. 2016. K-BOOM: a Bayesian approach to ontology structure inference, with applications in disease ontology construction [preprint]. bioRxiv. 10.1101/048843.

[iyaf215-B32] Musen MA, the Protégé Team. 2015. The protégé project: a look back and a look forward. AI Matters. 1:4–12. 10.1145/2757001.2757003. 27239556 PMC4883684

[iyaf215-B33] “NanbyoData.” n.d. NanbyoData. [accessed 2025 April 28]. https://nanbyodata.jp/?lang=en

[iyaf215-B34] “National Organization for Rare Disorders.” 2022. National Organization for Rare Disorders. March 8, 2022. https://rarediseases.org/

[iyaf215-B35] Nicholas F, Tammen I. 1995. Online Mendelian inheritance in animals (OMIA). University of Sydney. 10.25910/2AMR-PV70.

[iyaf215-B36] “OMIM .” n.d. [accessed 2025 April 18]. https://omim.org/

[iyaf215-B37] “Orphanet.” n.d. [accessed 2025 April 18]. https://www.orpha.net/

[iyaf215-B38] Osumi-Sutherland D, Courtot M, Balhoff JP, Mungall C. 2017. Dead simple OWL design patterns. J Biomed Semantics. 8:18. 10.1186/s13326-017-0126-0. 28583177 PMC5460348

[iyaf215-B39] Putman TE et al 2023. The Monarch Initiative in 2024: an analytic platform integrating phenotypes, genes and diseases across species. Nucleic Acids Res. 52:D938–D949. 10.1093/nar/gkad1082. PMC10767791 38000386

[iyaf215-B40] Richesson RL, Krischer J. 2007. Data standards in clinical research: gaps, overlaps, challenges and future directions. J Am Med Inform Assoc. 14:687–696. 10.1197/jamia.M2470. 17712081 PMC2213488

[iyaf215-B41] Rogers FB . 1963. Medical subject headings. Bull Med Libr Assoc. 51:114–116. https://pubmed.ncbi.nlm.nih.gov/13982385/. 13982385 PMC197951

[iyaf215-B42] Schriml LM et al 2022. The Human Disease Ontology 2022 update. Nucleic Acids Res. 50:D1255–D1261. 10.1093/nar/gkab1063. 34755882 PMC8728220

[iyaf215-B43] Sioutos N et al 2007. NCI thesaurus: a semantic model integrating cancer-related clinical and molecular information. J Biomed Inform. 40:30–43. 10.1016/j.jbi.2006.02.013. 16697710

[iyaf215-B44] Slebodnik M . 2009. Orphanet: the portal for rare diseases and orphan Drugs2009384Orphanet: the portal for rare diseases and orphan drugs. Institute National de La Santé et de La Recherche Médicale (INSERM). Last Visited June 2009. Gratis URL: Www.orpha.net/.” Reference Reviews. 10.1108/09504120911003492.

[iyaf215-B45] Stefancsik R et al 2023. The Ontology of Biological Attributes (OBA)-computational traits for the life sciences. Mamm Genome. 34:364–378. 10.1007/s00335-023-09992-1. 37076585 PMC10382347

[iyaf215-B46] Thaxton C et al 2022. Lumping versus splitting: how to approach defining a disease to enable accurate genomic curation. Cell Genom. 2:100131. 10.1016/j.xgen.2022.100131. 35754516 PMC9221396

[iyaf215-B47] Thaxton C et al 2024. Implementation of a dyadic Nomenclature for monogenic diseases. Am J Hum Genet. 111:1810–1818. 10.1016/j.ajhg.2024.07.019. 39241757 PMC11393707

[iyaf215-B48] Toro S et al 2024. Dynamic retrieval augmented generation of ontologies using artificial intelligence (DRAGON-AI). J Biomed Semantics. 15:19. 10.1186/s13326-024-00320-3. 39415214 PMC11484368

[iyaf215-B49] Tudini E et al 2022. Shariant platform: enabling evidence sharing across Australian clinical genetic-testing laboratories to support variant interpretation. Am J Hum Genet. 10.1016/j.ajhg.2022.10.006 PMC9674965 36332611

[iyaf215-B50] Vasilevsky N . 2022. “The Landscape of Disease and Phenotype Ontologies.” Zenodo. 10.5281/zenodo.6299898.

[iyaf215-B51] Vasilevsky N et al 2025. “Mondo Epilepsy Workshop Summary.” Zenodo. 10.5281/ZENODO.14894999.

[iyaf215-B52] Wagner AH et al 2021. The GA4GH variation representation specification: a computational framework for variation representation and federated identification. Cell Genom. 10.1016/j.xgen.2021.100027 PMC8929418 35311178

[iyaf215-B53] Wakap N et al 2020. Estimating cumulative point prevalence of rare diseases: analysis of the orphanet database. Eur J Hum Genet: EJHG. 28:165–73. 10.1038/s41431-019-0508-0. 31527858 PMC6974615

[iyaf215-B54] Wilkinson MD et al 2016. The FAIR guiding principles for scientific data management and stewardship. Sci Data. 3:160018. 10.1038/sdata.2016.18. 26978244 PMC4792175

[iyaf215-B55] “Workshop.” 2025. [2025 April 24]. https://mondo.monarchinitiative.org/pages/workshop/#november-2018

[iyaf215-B56] Wu J et al 2021. IDDB: a comprehensive resource featuring genes, variants and characteristics associated with infertility. Nucleic Acids Res. 10.1093/nar/gkaa753 PMC7779019 32941628

[iyaf215-B57] Xu F et al 2023. Features of a FAIR vocabulary. Biomed Sem. 10.1186/s13326-023-00286-8 PMC10236849 37264430

