# Supplementary material for: Mondo: integrating disease terminology across communities
Source: Genetics. 2025 Oct 6;232(4):iyaf215. doi: 10.1093/genetics/iyaf215 (PMC13050200; doi:10.1093/genetics/iyaf215)

Supplemental Figure 01: Number of unique community contributors by year between 2017 and 2024

The total number of unique community contributors excluding members of the Monarch Initiative and the Mondo core team.

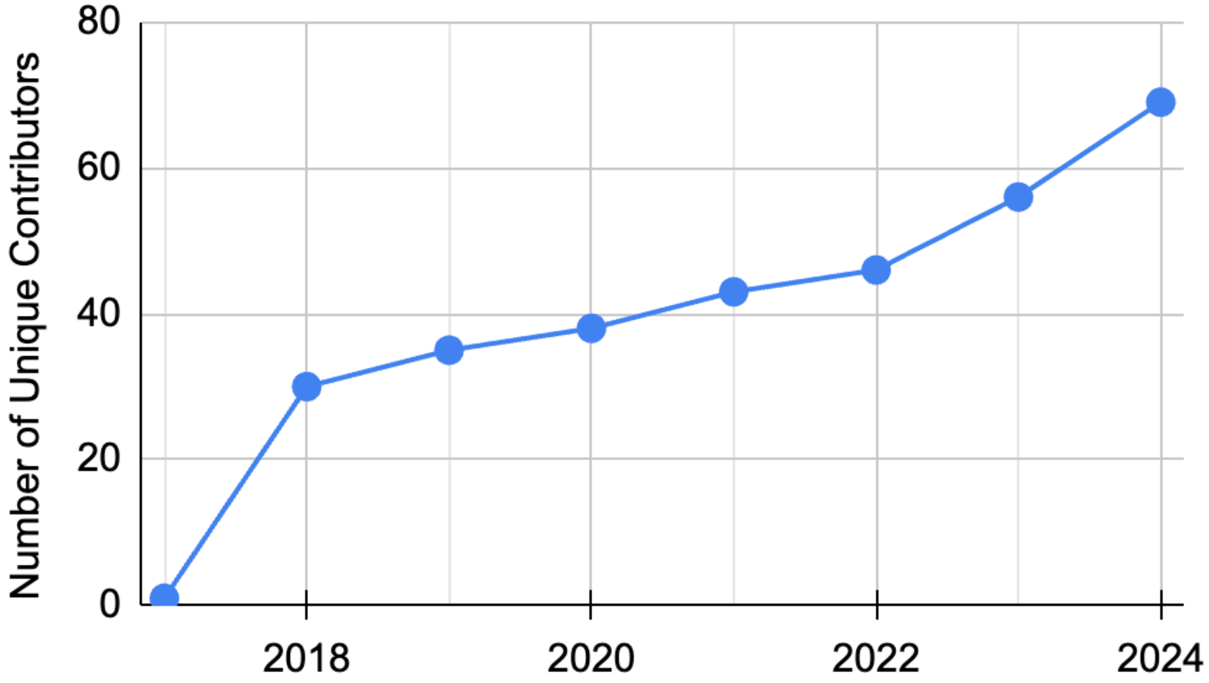

Supplement: iyaf215_Supplementary_Data [file iyaf215_supplementary_data.zip › Figure_S1_GENETICS-2025-308205.pdf]
