# Supplementary material for: Mondo: integrating disease terminology across communities
Source: Genetics. 2025 Oct 6;232(4):iyaf215. doi: 10.1093/genetics/iyaf215 (PMC13050200; doi:10.1093/genetics/iyaf215)
Supplement: iyaf215_Supplementary_Data [file iyaf215_supplementary_data.zip › Table_S1_GENETICS-2025-308205.pdf]

Supplemental Table 01: Major terminologies and key attributes represented in Mondo
Source ontologies for Mondo (https://mondo.monarchinitiative.org/pages/sources/).
Some ontologies/terminologies are integrated into Mondo in their entirety. Diseases in these resources serve as sources for Mondo disease concepts by providing labels, synonyms, definitions, and organization into a disease hierarchy. Other resources do not serve as sources, but are linked to using cross-references. This distinction (source concepts vs cross-references) is indicated in the 'Role' column.
1 source is authoritative for rare diseases
2 source is authoritative for Mendelian diseases
3 authoritative for cancer
4 non-human animal source

**Resource**	**ID Space / URI prefix**	**Role** source concepts vs cross-reference	**Website**
OMIM[2]	OMIM OMIMPS	source concepts	www.omim.org/
Orphanet[1]	ORDO Orphanet	source concepts	https://www.orpha.net/consor/cgi-bin/index.php
SNOMED (disorder subset)	SCTID	cross-references	http://www.snomed.org/
National Cancer Institute Thesaurus (NCIt, limited to human and non-human neoplasms)[3]	NCIT	source concepts	https://ncit.nci.nih.gov/ncitbrowser/
Genetic and Rare Diseases Information Center[1]	GARD	cross-references	https://rarediseases.info.nih.gov/
Medical Subject Headings	MESH	cross-references	https://id.nlm.nih.gov/mesh/
Unified Medical Language System	UMLS	cross-references	https://www.nlm.nih.gov/research/umls/index.html
ICD - ICD-9 - International Classification of Diseases	ICD9	cross-references	https://www.cdc.gov/nchs/icd/icd9.htm
ICD - ICD-10 - International Classification of Diseases	ICD10CM ICD10WHO	cross-references	https://www.cdc.gov/nchs/icd/icd10cm.htm
ICD 11 Foundation	icd11.foundation	cross-references (automatically matched)	https://icd.who.int/dev11/f/en
Experimental Factor Ontology	EFO	cross-references	https://www.ebi.ac.uk/efo/
Disease Ontology	DO	source concepts	http://www.obofoundry.org/ontology/doid.html
Mental Functioning Ontology	MF	cross-references	http://www.obofoundry.org/ontology/mf.html
MedGen	MEDGEN	cross-references	https://www.ncbi.nlm.nih.gov/medgen/
Ontology for General Medical	OGMS	cross-references	https://github.com/OGMS/ogms

Science			
MeDRA	MedDRA	cross-references	https://www.meddra.org/
OncoTree	ONCOTRE E	cross-references	http://oncotree.mskcc.org/#/home
NORD[1]	NORD	cross-references	https://rarediseases.org/
NANDO	NANDO	cross-references	https://nanbyodata.jp/about_nando
Online Mendelian Inheritance in Animals (OMIA)[2,4]	OMIA	source concepts (manually aligned)	https://omia.org/
