# Supplementary material for: Mondo: integrating disease terminology across communities
Source: Genetics. 2025 Oct 6;232(4):iyaf215. doi: 10.1093/genetics/iyaf215 (PMC13050200; doi:10.1093/genetics/iyaf215)
Supplement: iyaf215_Supplementary_Data [file iyaf215_supplementary_data.zip › Table_S2_GENETICS-2025-308205.pdf]

Supplemental Table 02: Synonyms

Total number of synonyms across all of Mondo, including diseases, injuries, disease susceptibilities and disease characteristics.

Synonym Type	Definition of Synonym Type	Example	Total number of synonyms	Total number of sources for synonyms
Exact	The definition of the synonym is exactly the same as the primary term label and definition. This is used when the same class can have more than one name.	‘familial Wilms' tumor’ is an exact synonym for hereditary Wilms' tumor’ (MONDO:0003321)	74,726	39
Narrow	The synonym is more specific or more narrow than the primary label and definition.	‘exercise-induced asthma’ is a narrow synonym for ‘asthma’ (MONDO:0004979)	2,544	20
Broad	The primary definition accurately describes the synonym, but the definition of the synonym may encompass other structures as well. In some cases where a broad synonym is given, it will be a broad synonym for more than one ontology term.	‘autoimmune liver disease’ is a broad synonym for ‘autoimmune hepatitis’ (MONDO:0016264)	1,368	22
Related	This scope is applied when a word or phrase has been used synonymously with the primary term name in the literature, but the usage is not strictly correct. That is, the synonym,, in fact has a slightly different meaning than the primary term name. Since users may not be aware that the synonym was being used incorrectly when searching for a term, related synonyms are included.	‘sudden unexpected nocturnal death syndrome’ is a related synonym for ‘Brugada syndrome’ (MONDO:0015263)	30,751	28
