# Supplementary material for: Mondo: integrating disease terminology across communities
Source: Genetics. 2025 Oct 6;232(4):iyaf215. doi: 10.1093/genetics/iyaf215 (PMC13050200; doi:10.1093/genetics/iyaf215)
Supplement: iyaf215_Supplementary_Data [file iyaf215_supplementary_data.zip › Table_S3_GENETICS-2025-308205.pdf]

Supplemental Table 03: Relationships
Select properties used in Mondo to describe relationships between diseases and other biomedical entities. These properties are used to define disease concepts in Mondo.

**Subject (ontology)**	**Relationship**	**Object (ontology)**	**Equivalence axioms examples**
disease (Mondo)	'disease has location'	Anatomical structure (UBERON)	Class: nervous system disorder (MONDO:0005071)<br>Axiom: 'disease or disorder' and ('disease has location' some 'nervous system')
disease (Mondo)	‘disease causes disruption of’	Biological process (GO)	Class: demyelinating disease (MONDO:0002562)<br>Axiom: 'neurodegenerative disease' and ('disease causes disruption of' some 'myelin maintenance')
disease (Mondo)	‘disease has feature’	Phenotype (HPO)	Class: paralytic poliomyelitis (MONDO:0000341)<br>Axiom: poliomyelitis and ('disease has feature' some Paralysis)
disease (Mondo)	'realized in response to'	Environmental exposure (ECTO)	Class: photokeratitis (MONDO:0001760)<br>Axiom: keratitis and ('realized in response to' some 'exposure to ultraviolet radiation')
disease (Mondo)	‘disease has basis in dysfunction of’	Gene (HGNC)	Class: Meckel syndrome, type 2 (MONDO:0011296)<br>Axiom: 'Meckel syndrome' and ('disease has basis in dysfunction of' some TMEM216)
disease (Mondo)	'disease arises from structure'	chromosome or chromosomal region (MONOCHROM)	Class: chromosome 16q22 deletion syndrome (MONDO:0013798)<br>Axiom: 'syndrome caused by partial chromosomal deletion' and ('disease arises from structure' some 'chr16q22 (Human)')
disease (Mondo)	‘disease has infectious agent’	Taxon (NCBITaxon)	Class: varicella zoster infection (MONDO:0005608)<br>Axiom: 'infectious disease' and ('disease has primary infectious agent' some 'Human alphaherpesvirus 3')
